# Rhein exhibits antioxidative effects similar to Rhubarb in a rat model of traumatic brain injury

**DOI:** 10.1186/s12906-017-1655-x

**Published:** 2017-03-07

**Authors:** Xia Xu, Huiying Lv, Zian Xia, Rong Fan, Chunhu Zhang, Yang Wang, Dongsheng Wang

**Affiliations:** 1 Laboratory of Ethnopharmacology, Institute of Integrated Traditional Chinese and Western Medicine, Xiangya Hospital, Central South University, 87 Xiangya Road, Changsha, 410008 People’s Republic of China; 2 Hunan Agricultural Product Processing Institute, Hunan Academy of Agricultural Sciences, and Hunan Food Test and Analysis Center, Changsha, 410125 People’s Republic of China

**Keywords:** Rhein, Rhubarb, Traumatic brain injury, Oxidative stress, Neuroprotective effect

## Abstract

**Background:**

The brain is secondarily harmed by pathological, physiological, and biological reactions that are caused by traumatic brain injury (TBI). Rhein, a significant composition of Rhubarb, is a well-known traditional Chinese treatment method and has a strong oxidation-resisting characteristic, but Rhein’s mechanism remains unclear.

**Methods:**

This study aimed to identify Rhein in the brain tissues of TBI model of rats, and confirm whether Rhein induced an antioxidative effect similar to its parent medicine, Rhubarb. First, the ultra performance liquid chromatography-tandem mass spectrometry (UPLC-MS/MS) method was applied to identify Rhein in the brain tissue of the controlled cortical impact (CCI) rats after intra-gastric administration of Rhubarb. Further, for the purpose of calculating the oxidant stress of the CCI rats, the malondialdehyde (MDA), catalase (CAT), superoxide dismutase (SOD), and glutathione disulfide (GSSG), as well as the proportion of glutathione (GSH)/GSSG were measured in the brain tissues.

**Results:**

The results showed that Rhein was absorbed in the brain tissues of CCI rats. Rhubarb and rhein elevated the SOD, CAT activities, GSH level, and GSH/GSSG ratio, and diminished the MDA and GSSG levels.

**Conclusion:**

The data demonstrated that Rhubarb and Rhein had the potential to be used as a neuroprotective drug for TBI, and that Rhein induced an antioxidative effect similar to its parent medicine, Rhubarb.

## Background

Traumatic brain injury (TBI) is a life-threatening disease worldwide [1]. Since the year 2000, more than 2 million Americans are affected by TBI each year and more than 70 billion US dollars are expended directly or indirectly [2]. Most Chinese people suffer from TBI due to injuries sustained at the sport grounds during exercise, or accidental falls in the office or at home, as well as motorcycle injuries [3]. TBI is called the “silent epidemic” [4] because of society’s lack of understanding about the condition.

Secondary brain injury following TBI triggers pathological, physiological, and biological reactions that result in brain malfunctions [5]. It often induces chain reactions in the biological and chemical processes, and inflammation, lack of energy by the cells, excitotoxicity, apoptosis, and oxidant stress may be caused as a result [6]. Among the previously mentioned processes, oxidative stress plays a pivotal role [7]. The promotion of inflammation and other reactions leads to the exacerbation of oxidative stress because oxidation aggravates inflammation and other reactions, which is a vicious cycle [8]. Oxidative stress occurs 1 h after TBI, becoming the pathological reaction that is immediately started [9]. Therefore, antioxidant strategy becomes the key in TBI treatment during the acute phase [10].

The brain is susceptible to oxidant injury, which is evidenced by the easiness in which the neuronal membrane [11] is peroxidized. Due to the abundant fatty acids, which can be easily peroxidized, the brain tissues need excessive oxygen, a proportion of 20% of the overall oxygen consumption compared with the 2% small weight percentage of the brain. The brain is rich in lipids, hence it will be more easily injured by the free radicals [12]. Reactive oxygen species (ROS), including superoxide anion, the hydroxyl radicals, and hydrogen peroxide (H_2_O_2_), have been recognized as specific second messengers in signaling cascades involved in cell growth and differentiation [13]. If ROS production and elimination of are not balanced, oxidative stress in the biological system will explode [14]. Excessive ROS generation is a vital factor contributing to oxidative stress in TBI development [15]. H_2_O_2_, the superoxide anions, the hydroxyl radicals, and other ROS are produced by the cells in the aerobic environment [16]. These free radicals can interact with biomolecules such as DNA, carbohydrates, protein, and lipids and damage various cellular components [17]. The brain is loaded with catalase (CAT), superoxide dismutase (SOD), glutathione (GSH), and other antioxidant enzymes to prevent oxidative injury. Meanwhile, malondialdehyde (MDA) and glutathione disulfide (GSSG) lead to oxidative stress in the brain tissue [18]. Under pro-oxidant conditions, two GSH molecules donate one electron each and are converted into GSSG. The GSH/GSSG molar ratio is considered a powerful index of oxidative stress and disease risk [19].

Unfortunately, TBI’s therapeutic efficacy is far from satisfactory because its pathogenesis is driven by extremely complex and interactive mechanisms [20]. Western medicine antioxidant strategies have failed [21, 22]. Scientists started to seek the potential innovative botanical compositions from traditional Chinese medicines (TCM) for the antioxidative treatment. Previous studies reported that TCM were successfully used as antioxidants in the treatment of brain disease [23]_._ Rhubarb is an herb frequently used in the clinic for treating digestive system diseases, including hepatitis, constipation, gastric ulcer, etc. [24]. Currently, Rhubarb’s application has expanded to the field of many chronic illnesses, including cancers, atherosclerosis, inflammation, etc. [25].

Recently, Rhubarb has been shown has protective effects against brain disturbances induced by severe cerebral injury. Rhubarb is a species of plant in the family Polygonaceae. In the Chinese Pharmacopoeia (PPRC, 2010a), three species are assigned as official Rhubarb, i.e., Rheum palmatum L., Rheum tanguticum Maxim. ex Balf., and Rheum officeinale Baill [26]. It has been shown that Rhubarb inhibits lipid peroxidation in rat brain homogenates [27]. Treatment with Rhubarb significantly decreases lactate dehydrogenase release and DNA fragmentation, which is important in the process of cell apoptosis [28]. Rhubarb has been used in recent years to treat TBI and has achieved a satisfied efficacy [29]. Rhein, as a significant active part of Rhubarb, is rich in effective oxidation performances [30]. However, the details of mechanism to treat TBI remains unknown [31]. Thus, this study aimed to determine Rhein’s antioxidant effects and further identify whether Rhein could be used as the targeted efficacy material of Rhubarb in the treatment of TBI.

In summary, this study was performed to provide material evidence of the underlying antioxidant Rhein in the brain tissues of CCI rats after Rhubarb administration by ultra performance liquid chromatography-tandem mass spectrometry (UPLC-MS/MS) method. Subsequently, the antioxidant effects of Rhein and its parent medicine, Rhubarb, on TBI model of rats were measured by SOD, MDA, and CAT, as well as the GSH/GSSG ratio. We were strongly interested in the hypothesis that whether Rhein could induce antioxidative stress similar to its parent medicine, Rhubarb.

## Methods

### Plant materials and chemicals

The dried, raw material Rhubarb plants were purchased from Xiangya Hospital of Central South University’s pharmacy, the analysis gradient methanol for liquid chromatography was purchased from Merck Company, Inc. (Darmstadt, Germany), and the normative reference of Rhein, the purity by authorization >98%, was purchased from the National Institute for the Control of Pharmaceutical and Biological Products (Beijing, China). The triple distilled water and the formic acid were acquired from the quartz glass device in the lab for preparing the moving phase and the Sinopharm Chemical Reagent Company in Shanghai, respectively. The MDA, SOD, GSH, GSSG, CAT, and Bradford protein assay kits were obtained from Nanjing Jiancheng Bioengineering Institute (Nanjing, China). The remaining reagents adopted the analysis levels.

Crushed Rhubarb into small pieces, extracted Rhubarb by refluxing in boiling water for 15 min. Filtered the water extraction through a five-layered cotton bandage. Concentrated and lyophilized the filtrate and stored it under 4 °C. Filtered the samples through 0.22 μm nylon filter (Bio Basic Inc.,Canada) before UPLC analysis.

### Animals and surgical procedure

200 to 300 g of male Sprague-Dawley (SD) rats, which grew in Changsha City, China, were bred in the house with the standard condition, especially at 22 ± 2 °C, with a 12-h light and dark circulation from 6.30 a.m. to 6.30 p.m. and a relative humidity of 50 ± 10%. The Animal Care and Use Committee of Central South University approved all experiment processes and agreements after conducting examinations. The rats were not fed with food 12 h before the test, but their water supply was sufficient.

One hundred eight of SD rats were randomly divided into six groups for the efficacy experiment with 3 time points for each group (8,16 and 24 h): (1) Vehicle control group: rats with TBI were intragastrically given the same amount of normal saline vehicle (0.9% NaCl) (*n* = 6); (2) sham operation group: rats underwent the same surgical procedures except that the cerebral cortex had not experienced trauma (*n* = 6); (3) 12 g/kg of Rhubarb treatment group: rats were orally given Rhubarb after the same trauma was performed (*n* = 6); (4) 6 g/kg of Rhubarb group: rats were orally given Rhubarb after the same trauma was performed (*n* = 6); (5) 3 g/kg of Rhubarb group: rats were orally given Rhubarb after the same trauma was performed (*n* = 6); (6) 12 mg/kg of Rhein group: rats were orally given Rhein after the same trauma was performed (*n* = 6).

The controlled cortical impact (CCI) model rats with TBI were performed as previously described with some modifications [32]. In short, we adopted 3% pentobarbital to anesthetize the male SD rats before locating them behind an air hammer through the Knopf three-dimensional frame, wherein the air hammer was an accurate scientific tool, TBI-0310. Then we operated on one side of the central gap at the right portion of the skull, which was located at the center between the bregma and the lambda with a 5-mm craniotomy aided by a handheld trepan, thus harming only one side of the experimental rats’ brain surface. Among the damage indexes, there was a bumping injury as deep as 5 mm lasting for 500 ms at 6 meters/second. We also performed a craniotomy on the control group rats but did not hammer the rats’ brains. Then we sutured the wounds and woke up the rats, placing them on a heating cushion so that the normal body temperature could be kept for 30 to 60 min. We observed the injured rats for at least 4 h every day.

### The Rhein in brain tissue from CCI Rat was detected by the UPLC-MS/MS method

Following the oral administration of Rhubarb to the CCI rats, brain tissue absorption of the compound was identified by comparing their ion peaks and retention times with the authentic reference using the UPLC-MS/MS method.

The Waters Acquity UPLC^TM^ system (Waters, Milford, MA) coupled to a Waters TQD triple quadruple tandem mass spectrometer was used to perform UPLC-MS/MS analysis. Acquity UPLC BEH 2.1 × 50 mm id, 1.7 μm C_18_ column using an Acquity UPLC system equipped with an Acquity photo-diode array detector was used to perform chromatographic separation. The mobile phase consisted of methanol/deionized water in which the column had 0.1% formic acid with a gradient elution (0 min, 45:55; 15 min, 75:25). The UPLC detection parameters were as follows: the column oven temperature was set at 35 °C; the injection volume was 5 μL, using a flow rate of 0.25 mL/min; and the ultraviolet (UV) spectrum wavelength was 254 nm. The MS/MS detection parameters were as follows: the temperature of the anti-solvent gas (nitrogen) flow of 650 L/h was at 365 °C, the temperature of the source gas (nitrogen) was 110 °C, the cone gas flow was 50 L/h, the collision gas (argon) flow was 0.2 mL/min, and the capillary voltage was 2.5 KV. During the inspection, the multiple reaction monitoring (MRM) method was adopted.

The Rhubarb (3 g/kg, 6 g/kg, 12 g/kg) and Rhein (12 mg/kg) were separately gavaged to CCI rats, each group of rats were sacrificed at 8 h, 16 h and 24 h after intragastric administration. Rats were anesthetized with an intra-peritoneal injection of 10% chloral hydrate at a dose of 400 mg · kg^−1^. The whole brain was harvested 30 min after decapitation. The sample was thawed to room temperature before analysis. Brain tissue samples were briefly washed in Adequate ice-cold dd H_2_O, then homogenized by the addition of 4 mL ice-cold methanol used a TissueLyser LT homogenizer (QIAGEN, Hilden, Germany). The homogenates were centrifuged at 3000 rpm at 4 °C for 10 min. After centrifugation, they were evaporated to dryness and the supernatant was separately collected with nitrogen at 37 °C. Two hundred μL of 20% methanol was used to dissolve the dry extract, and then the solution was centrifuged at 15000 rpm at 4 °C for 15 min. The upper layer was filtered with a 0.22-μm nylon filter after centrifugation. The filter (5 μL) was injected into the UPLC-MS/MS system for analysis.

The Waters Acquity™ TQD triple quadruple tandem mass spectrometer installed with an ESI interface was connected to the UHPLC system for operation in MS/MS mode. Data acquisition and processing were done with Masslynx™ 4.1 software. The UPLC conditions were the same as those for the UPLC-DAD analysis.

### Estimation of oxidative and antioxidative status

The stored cortexes were weighed, dissected, and homogenized by a homogenizer (TissueLyser LT, German) with 9 volumes (1:9, w/v) of ice-cold normal saline. The homogenates were centrifuged at 3000 rpm at 4 °C for 15 min. The antioxidant enzyme activities, oxidative product contents, and redox status of the supernatants were measured according to the assay kits’ illustrations (Nanjing Jiancheng Institute of Biological Engineering). Tissue protein concentrations were measured following the Bradford method.

#### Using the Bradford method to measure the superoxide dismutase activity

The total SOD activity procedures followed the assay kits’ instructions (Nanjing Jiancheng Institute of Biological Engineering). The test liquid’s absorption rate was measured at a wavelength of 450 nm. The activities were corrected with protein amount and were expressed as fold of control. Absorbance was read at 450 nm and the activity of SOD was calculated using the formula: [(control value-blank value)-(sample value-blank value)]/(control value-blank value) × 2 × (total volume/sample volume)/protein Concentration. The U/mg proteins were adopted to represent the SOD activity.

#### Measuring the activity of the catalase

CAT activity was assayed by the assay kits’ instructions (Nanjing Jiancheng Institute of Biological Engineering). Incubated the samples with excess hydrogen peroxide for 10 min. Then coupled the hydrogen peroxide with a substrate to produce N-4-antipyryl-3-chloro-5-sulfonate-p-benzoquinonemonoimine by peroxidase treatment. The test liquid’s absorption rate was measured at a wavelength of 450 nm. The μmol H_2_O_2_-consumed/min/mg proteins were used to represent the enzymes’ activity.

#### Assay of malondialdehyde levels

For the MDA measurement, we used a commercial kit (Beyotime Institute of Biotechnology, Suzhou, China) to quantify the generation of MDA according to the manufacturer’s protocol. To determine the MDA levels, we used the thiobarbituric acid (TBA) method, which Hui Zhao et al. described. According to the instructions, the TBA and the supernatant were mixed together and incubated at 95 °C for 40 min. The reaction mixture was cooled to room temperature by flowing water and centrifuged at 3500 rpm for 10 min. Then the supernatant was used to test the relative MDA units through a Tecan Safire 5 microplate reader (532 nm). The results were expressed as the contents μmol per mg protein.

#### Determination of glutathione activity and oxidized glutathione levels

The GSH activity and oxidized GSSG protocols followed the GSH/GSSG assay kits’ specifications (Jiancheng Institute of Biological Engineering as reported by Hui Zhao et al. Glutathione (GSH) was determined based on added 5,5-dithiobis-2-nitrobenzoic acid (DTNB) to compounds containing sulfhydryl groups developing a yellow color. The absorbance was recorded at 405 nm in a spectrophotometer after 10 min, then the GSH/GSSG ratio was calculated. Total GSH content was expressed as μmol/mg protein.

### Statistic analysis

SPSS 15.0 software (SPSS Inc., Chicago, IL) was used to analyze all the data. All parameters were expressed as the mean ± standard deviation (SD). Turkey as post hoc test was used to determine the significant differences between the four groups. Values of *p* <0.05 were considered statistically significant.

## Results

### Rhein detection by UPLC-MS/MS

**Fig. 1:**
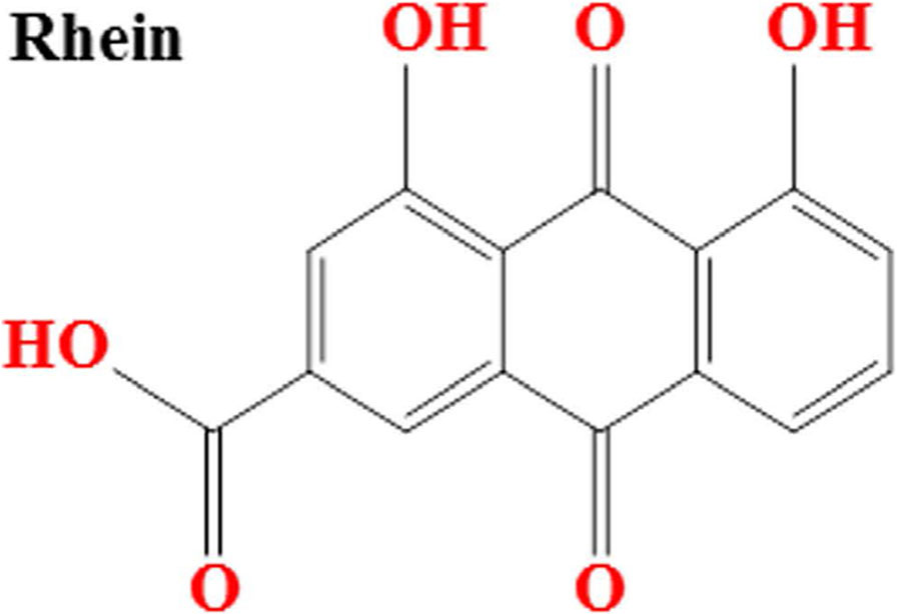
The chemical structure of Rhein (4, 5-dihydroxyanthraquinone-2-carboxylic acid). Molecular formula: C15H8O6. Molecular weight: 284.22 g/moL

**Fig. 2:**
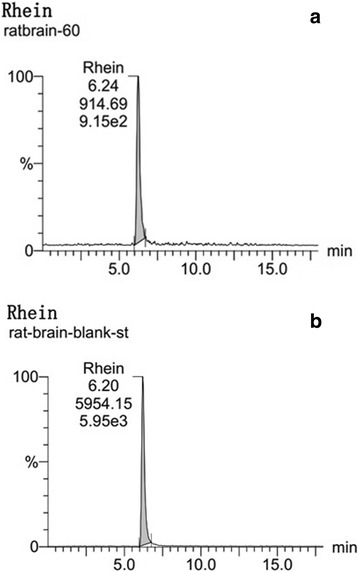
Identification of Rhein in the brain tissue of a CCI rat by Ultra-high Performance Liquid Chromatography with tandem mass spectrometry (UPLC-MS/MS) analysis. Representative MRM chromatograms of Rhein in brain sample. **a** Brain sample detection at 1 h after intragastric administration of Rhubarb (12 g/kg); (**b**) Blank brain tissue spiked with reference Rhein

In this study, an UPLC-MS/MS method was successfully developed to analyze Rhein in the brain tissue of CCI rats after administration of Rhubarb. The intra-cerebral absorption component is Rheum officinale anthraquinone component Rhein, the Chemical Structure was showed in Fig. 1. The overall intra- and inter-day variations were <5% for Rhein. The method was reproducible and sufficiently sensitive. Recovery test and sample analysis showed the tests’ accuracy. The recovery of all tested compounds was more than 90%. All results showed that the development and validation purpose of the UPLC-MS/MS method was to detect Rhein. The MS/MS predominated mass transitions were m/z 283.01 → 239.0 for Rhein. The precursor ions were m/z 283.01 [M-H]-. The m/z 239.0 was the prominent fragment ion in the production mass spectra of [M-H]-. Rhein was explicitly defined in biopsies or extracts based on the fragment ions (m/z 239.0), the retention time (6.32 ± 0.08 min), and the characteristics [M-H]-ions (m/z 283.01). Fig. 2 showed the typical multiple reaction monitoring (MRM) chromatogram using UPLC-MS/MS analysis. According to the chromatography, we found that Rhein was identified in brain tissue of CCI rat.

### Effects of the antioxidant assay

#### Rhubarb and Rhein significantly increased SOD level in the brain tissue of CCI rats

**Fig. 3:**
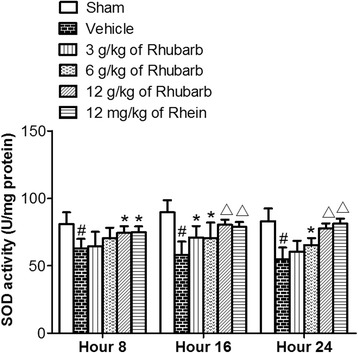
Effects of Rhubarb (3 g/kg, 6 g/kg and 12 g/kg) and Rhein (12 mg/kg) on the SOD levels of rat brains at different time points. Values are expressed as the mean ± SD. ^#^ *p* < 0.01 compared to the sham at the same time point, *p < 0.05 compared to the vehicle at the same time point, ^△^ *p* < 0.01 compared to the vehicle at the same time point

Compared with the sham treatment, CCI rats gave rise to a significant decline of SOD activities (^#^ *p* < 0.01) (Fig. 3). The administration of Rhubarb (12 g/kg) and Rhein (12 mg/kg) caused a significant increase in SOD levels at different time points (16th and 24th hours) compared with the vehicle treatment(^△^ *p* < 0.01). The application of Rhubarb (6 g/kg) gave rise to a significant increase in the SOD levels at different time points (16th and 24th hours) compared with the vehicle treatment (*p < 0.05). The application of Rhein (12 mg/kg) increased the SOD levels at 8 h compared with the vehicle treatment (*p < 0.05). The administration of Rhubarb (3 g/kg and 6 g/kg) at the 8th hour had no statistical significance (*p* > 0.05) when compared with the vehicle treatment.

#### Rhubarb and Rhein evidently increased CAT level in the brain tissue of CCI rats

**Fig. 4:**
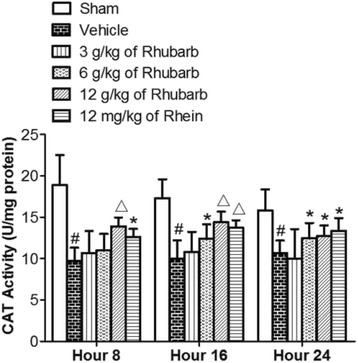
Effects of Rhubarb (3 g/kg, 6 g/kg and 12 g/kg) and Rhein (12 mg/kg) on the CAT levels of rat brains at different time points. Values are expressed as the mean ± SD. ^#^ *p* < 0.01 compared to the sham at the same time point, *p < 0.05 compared to the vehicle at the same time point, ^△^ *p* < 0.01 compared to the vehicle at the same time point

Compared with the sham treatment, CCI rats gave rise to a significant decline in CAT activities (^#^ *p* < 0.01) (Fig. 4). Fig. 4 shows that CAT activities increased significantly at the 8th and 16th hours after a high dose of Rhubarb (12 g/kg), as well as at the 16th hour after a dose of Rhein (12 mg/kg) compared with the vehicle treatment(^△^ *p* < 0.01). The application of Rhein (12 mg/kg) increased the CAT levels at different time points (8th and 24th hours) compared with the vehicle treatment (*p < 0.05). The administration of Rhubarb (3 g/kg) had no statistical significance at all time points (8th, 16th and 24th hours) compared with the vehicle treatment (*p* > 0.05).

#### Rhubarb and Rhein significantly decreased MDA level in the brain tissue of CCI rats

**Fig. 5:**
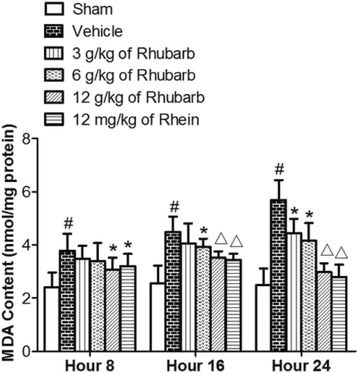
Effects of Rhubarb (3 g/kg, 6 g/kg and 12 g/kg) and Rhein (12 mg/kg) on the MDA levels of rat brains at different time points. Values are expressed as the mean ± SD. ^#^ *p* < 0.01 compared to the sham at the same time point, *p < 0.05 compared to the vehicle at the same time point, ^△^ *p* < 0.01 compared to the vehicle at the same time point

As shown in Fig. 5, significantly more MDA levels were measured in the brains of CCI rats compared with the sham group (^#^ *p* < 0.01). Fig. 5 shows that MDA activities significantly decreased at the 16th and 24th hours after a high dose of Rhubarb (12 g/kg) and Rhein (12 mg/kg) compared with the vehicle treatment(^△^ *p* < 0.01). The application of Rhubarb (6 g/kg) significantly decreased the MDA levels at different time points (16th and 24th hours) as well as Rhein (12 mg/kg) at the 8th hour compared with the vehicle treatment (*p < 0.05). The administration of Rhubarb (3 g/kg and 6 g/kg) had no statistical significance at the 8th hour compared with the vehicle treatment (*p* > 0.05). The maximum value alteration occurred at the 24th hour.

#### Rhubarb and Rhein evidently increased GSH levels and GSH/GSSG ratios in the brain tissue of CCI rats

Brain injury in CCI rats led to reduced GSH levels and GSH/GSSG ratios, and simultaneously increased GSSG levels (all *p* < 0.01). As shown in Table 1, the treatments of Rhubarb (3, 6, and 12 g/kg) and Rhein (12 mg/kg) significantly increased the GSH levels and GSH/GSSG ratios and decreased the GSSG levels in the brain tissues of CCI rats compared with the vehicle group.

**Table 1:** The effect of rhubarb its absorbed compound rhein on brain tissues of TBI rats in reduced and oxidized glutathione levels

	8 Hour			16 Hour			24 Hour		
Group	GSH (μmol/L	GSSG (μmol/L)	GSH/GSSG	GSH (μmol/L)	GSSG (μmol/L)	GSH/GSSG	GSH (μmol/L)	GSSG (μmol/L)	GSH/GSSG
Sham	100.23 ± 7.09	27.29 ± 3.54	3.67 ± 0.65	95.57 ± 8.54	26.98 ± 2.14	3.54 ± 0.33	97.24 ± 8.03	29.34 ± 1.88	3.31 ± 0.29
Vehicle	70.12 ± 6.95^#^	50.61 ± 4.61^#^	1.39 ± 0.11^#^	69.22 ± 7.17^#^	52.14 ± 3.67^#^	1.33 ± 0.09^#^	62.71 ± 6.24^#^	48.87 ± 3.96^#^	1.28 ± 0.22^#^
3 g/kg Rhubarb	71.17 ± 6.25	42.81 ± 4.77^*^	1.66 ± 0.14^*^	75.97 ± 6.25^*^	49.36 ± 4.02	1.54 ± 0.22	74.02 ± 7.14^△^	39.57 ± 4.43^*^	1.87 ± 0.29^*^
6 g/kg Rhubarb	72.14 ± 7.47	38.21 ± 3.31^△^	1.89 ± 0.17^△^	79.64 ± 8.24^*^	48.74 ± 3.49^*^	1.63 ± 0.16^*^	83.81 ± 8.16^△^	32.88 ± 2.82^△^	2.55 ± 0.27^△^
12 g/kg Rhubarb	85.62 ± 7.15^△^	35.78 ± 4.11^△^	2.39 ± 0.27^△^	89.27 ± 8.07^△^	33.96 ± 3.51^△^	2.63 ± 0.29^△^	92.55 ± 8.43^△^	31.34 ± 2.5^△^	2.95 ± 0.32^△^
12 mg/kg Rhein	78.69 ± 7.06^△^	34.28 ± 3.84^△^	2.30 ± 0.25^△^	93.74 ± 8.84^△^	41.44 ± 3.87^△^	2.26 ± 0.18^△^	90.87 ± 8.31^△^	35.35 ± 3.47^△^	2.57 ± 0.18^△^

Each value represents mean ± SD (*n* = 6). Sham group (shame-operated control), Vehicle group (orally given vehicle), 10 mg/kg HSYA (orally given 10 mg/kg HSYA) and 30 mg/kg HSYA (30 mg/kg orally given HSYA). ^#^ *p* < 0.01 as compared to sham, ^△^ *p* < 0.01 as compared to vehicle and ^*^ *p* < 0.05 as compared to vehicle

## Discussion

This study aimed to identify Rhein in the brain tissues of CCI rats and confirm whether Rhein induced an antioxidative effect similar to its parent medicine, Rhubarb. The present study demonstrated that Rhein was absorbed into the brain tissues of rats with TBI after gavage administration of Rhubarb using UPLC-MS/MS method. Rhubarb and Rhein significantly increased the SOD, CAT activities, GSH level, and GSH/GSSG ratio. Meanwhile, Rhubarb and Rhein evidently decreased the MDA and GSSG levels. Rhein played a similar role as Rhubarb via the antioxidant pathway. The results suggested that Rhein could be used as the effective material basis of Rhubarb through antioxidation in TBI treatment.

The brain is particularly susceptible to attacks by oxidative stress after TBI because it is found to generate more toxicants per gram of tissue than any other organ [33]. Due to the abundant polyunsaturated fatty acids located in the membrane may be subject to lipid peroxidation easily, the brain is apt to get oxidative injury [34]. According to the previous research, the brain has a high oxidation metabolizing activity but a low antioxidative enzyme activity [35]. This imbalance leads to ischemic and toxic incidences, subsequently hurting the neurons easily. If the imbalance occurred in the antioxidant or pro-oxidant homeostasis, toxic ROS and the oxidative stress will be incurred [36]. The damage to the antioxidative system may hurt the neuro tissues and cells following TBI [37]. When oxidative stress burst out during TBI, the peroxidative enzymes will be activated, the phospholipid of the membranes and the functions of mitochondria will be damaged, and a lot of cell components such as RNAs, DNAs, lipids, sugars, and protein will be hurt. As a result, the activities of the cells may be influenced, and the brain may suffer further from the neurodegeneration [38].

To alleviate oxidative reaction after TBI, key enzymes associated with oxidative stress should be regulated. The most important enzymatic antioxidants contain CAT, SOD, and GSH, which prevent the brain from oxidative injury. When the superoxide is converted to hydrogen peroxide, the SOD has the effect of catalysis, while CAT prevents hydrogen peroxide from converting into oxygen and water [39]. The cells are rich in GSH, one of the major antioxidant compounds in body fluids that belong to thiol, with the smallest molecule weight [40]. MDA is the marker of oxidative lipid damage. The amount of MDA, a product of lipid peroxidation, is a measure of the cell or tissue’s oxidative stress status. As the oxidative stress content is increased while the antioxidation activity is reduced, the MDA concentration in the nervous tissues rose [41]. When cells are exposed to oxidative stress, reduced GSH will become GSSG during the process of binding free radicals. The GSH/GSSG measurement is a useful indicator of oxidative stress and could be used to monitor the antioxidant effect.

TCM has been used in China, Korea, Japan, and other Asian countries for the treatment of a wide range of diseases. Due to the heterogeneous property of TBI’s pathological physiology, the application of the multiple-drug combined treatment method in the multiple molecule targets was approved [42]. Therefore, TCM, which generally contains more than one ingredient and works at many sites, is accordant with the requirements of the possible TBI therapy [43]. In recent years, many reports were published about the TCM’s effects in improving the activity of the antioxidation defense enzymes and reducing or eliminating the generation of free radicals caused by the oxygen [44]. Accordingly, TCM has attracted more attention for its potential application in clinical research as well as in animal models [45]. Our study provided a canonical method to illuminate the mechanism of herbal chemicals and suggested that Rhein could be a promising therapeutic compound for TBI treatment.

A recent study confirmed that Rhubarb-originated Rhein can cross the blood-brain barrier(BBB) and reduce the BBB’s tight junction damage [46]. Consequently, Rhubarb was administered orally in many studies for TBI treatment [47]. The previous research demonstrated that Rhubarb protect BBB following TBI via an antioxidative molecular mechanism [48]. The accumulation of Rhein in the brain after Rhubarb administration following TBI could exert neuroprotection [49]. Therefore, benefitting from the BBB openness after TBI, Rhein had opportunities to produce the antioxidative effects by crossing the barriers [50].

After adopting the UPLC-MS/MS method, the resolution was improved and additional selectivity was provided. UPLC was a method with good precision, a high recovery rate, simple operation, innocuity, and good reproducibility [51]. It was a powerful analysis tool for complex samples, such as the component determination of TCM [52]. In this study, we detected Rhein in the brain tissues of rats with TBI after Rhubarb administration by the UPLC-MS/MS method, to establish a link between the absorbed chemical compound and its biological activity. If absorptive evidence did not exist, the study of an effect-related component was most likely incorrect. Thus, in this study, an accurate and sensitive UPLC-MS/MS method was successfully developed to detect Rhein in the brain tissues of rats with TBI after gavage administration of Rhubarb.

According to the Rhein’s mass spectrum (Fig. 2), the mass transitions that followed were m/z 283.01 → 239.0. The Rhein was identified by comparing the retention time and ion peaks in Fig. 2a and b. The results provided evidence that Rhein was absorbed into the brains of CCI rats after Rhubarb administration. The detection of the bioactive compound may provide material evidence for the Rhubarb’s pharmacological actions to treat TBI.

Rhubarb significantly improved SOD, CAT activities, GSH level, and GSH/GSSG ratio in the brain tissues of CCI rats. Meanwhile, Rhubarb evidently diminished the MDA and GSSG levels. Rhein played a similar role as Rhubarb above the antioxidant pathway. Rhein was the absorbed bioactive composition of Rhubarb to treat TBI. The previous data provided a rationale suggesting that Rhein induced antioxidant effect and played a similar role to Rhubarb in the treatment of TBI. Rhein and Rhubarb have the potential to be utilized as neuroprotective drugs for TBI.

The results suggested that Rhubarb and rhein attenuated the excessive formation of free radicals of oxidative stress in a rat model of TBI. Rhein exerted a similar role to its parent medicine Rhubarb in antioxidation. However, further study should be done to investigate the more details on molecular mechanisms, such as the potential signal pathway to confirm antioxidation of Rhein and its parent medicine, Rhubarb, for TBI treatment.

## Conclusion

Rhein was the bioactive component absorbed into the brains of a CCI rats after Rhubarb administration. Rhein played a similar effect to Rhubarb in the treatment of traumatic brain injury through antioxidation (via boosting the SOD, CAT activities, GSH level, and GSH/GSSG ratio and concomitantly diminishing MDA and GSSG levels). The results supported that Rhubarb and its absorbed component Rhein had the potential to be utilized as the neuroprotective drug for TBI treatment.

## Abbreviations

%: Percentage
°C: Degree Celsius
BBB: Blood-brain barrier
DNA: Deoxyribonucleic acid
H_2_O_2_: Hydrogen peroxide
mm: Millimeter
ms: Meters/second
RNA: Ribonucleic acid
rpm: Revolutions per minute
TBA: Thiobarbituric acid
UV: Ultra violet
μl: Microliter
